# Krill oil extract suppresses cell growth and induces apoptosis of human colorectal cancer cells

**DOI:** 10.1186/s12906-016-1311-x

**Published:** 2016-08-30

**Authors:** Abilasha Gayani Jayathilake, Paul Vincent Senior, Xiao Qun Su

**Affiliations:** 1 Centre for Chronic Disease, College of Health and Biomedicine, Victoria University, P.O. Box 14428, Melbourne, VIC 8001 Australia; 2 Melbourne Medical School Western Campus, Western Centre for Health Research and Education, Sunshine Hospital, St Albans, VIC 3021 Australia

**Keywords:** Krill oil extract, Eicosapentaenoic acid, Docosahexaenoic acid, Human colorectal cancer cells

## Abstract

**Background:**

Colorectal cancer (CRC) is the third most common cancer in the world. The current available treatments for CRC include surgery, chemotherapy and radiotherapy. However, surgery is only useful when the disease is diagnosed at the earlier stage. Chemotherapy and radiotherapy are associated with numerous side effects that decrease the patients’ quality of life. Safer, effective alternatives, such as natural compounds, to chemotherapy are desirable. This study assessed the efficacy of free fatty acid (FFA) extract of krill oil on three human CRC cells lines.

**Methods:**

HCT-15, SW-480 and Caco-2 cells were treated with the FFA extracts of krill oil and fish oil for 48 h while treatments with the bioactive omega-3 polyunsaturated fatty acids (LC n-3 PUFA) of these marine oils, eicosapentaenoic acid (EPA, C20:5n-3) and docosahexaenoic acid (DHA, C22:6n-3) in comparison with a n-6 PUFA, arachnoid acid (AA, C20:4n-6) were up to 72 h at the concentrations of 50, 100, 150 and 200 μM. Effects of all the treatments on cell proliferation were assessed using a water-soluble tetrazolium-1 (WST-1) assay kit at 24, 48 and 72 h. Effects of FFA extract of krill oil and EPA on apoptosis and mitochondrial membrane potential were determined using commercial kits after 48 h of treatment.

**Results:**

Krill oil extract inhibited cell proliferation of all three cell lines in the similar manner as fish oil extract. A significant cell apoptosis and increase in mitochondrial membrane potential were observed after the treatment with krill oil extract. EPA at the concentration of 200 μM reduced significantly the proliferation of HCT-15 and SW-480 at 24, 48 and 72 h. In addition, EPA treatment (100 and 200 μM) resulted in significant cell apoptosis in all three cell lines. No significant changes were observed after treatment with DHA and AA.

**Conclusions:**

Our results indicate that the FFA extract of krill oil maybe an effective chemotherapeutic agent to suppress proliferation and induce apoptosis in CRC cells through its bioactive constitute EPA. Although the exact mechanism of the pro-apoptotic properties of krill oil extract is unclear, mitochondrial pathway seems to be implicated.

## Background

Colorectal cancer (CRC) is the third most common cancer in men and women, accounting for approximately 9 % of mortality caused by cancer each year [1, 2]. The initiation of CRC is a complex and multifactorial process that is associated with progressive accumulation of genetic and epigenetic alterations, and these transform normal colonic/rectal mucosa into invasive metastatic carcinoma [3, 4]. Among many factors associated with the development of colorectal cancer, more than 70 % of CRC patients diagnosed are sporadic, and environmental factors have been the main cause for the majority of incidence [3]. Diet is one of the main environmental factors, with 90 % of the CRC reported to be associated with high intake of saturated fat, red meat, n-6 polyunsaturated fatty acids (PUFA), and low intake of fibres and vitamins [5].

The current available treatments for CRC include surgery, chemotherapy and radiotherapy [3, 6]. However, surgery is only curative when the disease is diagnosed at an earlier stage. Chemotherapy and radiotherapy are associated with numerous side effects, such as myelosuppression, mucositis, dermatitis and diarrhoea, thus these treatments have a significant impact on patient's quality of life [6, 7]. In recent years, the potential role of nutrients as preventive/therapeutic agents has been one of the main foci in cancer research [6–8].

Fish oil is a rich source of long chain omega-3 polyunsaturated fatty acids (LC n-3 PUFA), mainly eicosapentaenoic acid (EPA, C20:5n-3) and docosahexaenoic acid (DHA, C22:6n-3). EPA and DHA have demonstrated preventive and therapeutic effects on the CRC [9, 10]. Epidemiological studies showed that increased consumption of fish oil or LC n-3 PUFA is inversely correlated with CRC incidence [11, 12]. Studies have shown that LC n-3 PUFA have growth-inhibitory and pro-apoptotic effects on colon cancer cells such as HT-29, HCT-116, SW-480 and Caco-2 [13–15]. Animal studies showed that fish oil supplementation reduced the number and size of polyps and suppressed the growth of cancer xenografts in nude mice [16–20].

Several sustainable alternatives of fish oil have been identified to meet the demand for LC n-3 PUFA during last decades and one of these is the krill oil. Krill oil is extracted from *Euphasia superba*, a crustacean species similar to shrimp found in the Southern Ocean [8]. Apart from the LC n-3 PUFA, EPA and DHA, krill oil also contains astaxanthin (provitamin E), flavonoids and vitamin-A. Different from fish oil, in the krill oil, EPA and DHA are bound to the phospholipids [21, 22]; while in the fish oil they are mainly bound to the triglycerides [23]. Studies have shown that the phospholipids bound n-3 PUFA can penetrate through cell membrane efficiently [24] than those bound to glycerol in triglycerides thus could potentially lead to better health outcomes [8, 25].

Preliminary studies have shown that krill oil has anti-cancer properties such as inhibition of proliferation of osteosarcoma and colon cancer SW-480 cells [8, 26]. However, little information is available on the effect of krill oil on other CRC cells, and no data are available on its impact on cell apoptosis. Apoptosis is a key process of programmed cell death, which plays a crucial role in maintaining cellular homeostasis between cell division and cell death [27]. Alterations in the mitochondrial membrane potential (MMP) and permeability could trigger the release of cytochrome *c* into the cytosol, which activates caspases that in turn, induce apoptosis [11]. In the present study, we investigated the effects of free fatty acid (FFA) extract from krill oil in comparison with that from fish oil on three human CRC cell lines HCT-15, SW-480 and Caco-2. In addition, effects of EPA and DHA on these cells are also assessed. To the best of our knowledge, this is the first study assessing the impacts of krill oil on cell apoptosis and investigating whether the apoptotic process is mediated by changes in MMP.

## Methods

### Cell lines and culture conditions

The human colon adenocarcinoma cell lines of HCT-15, SW-480 and Caco-2 were obtained from the American Tissue Culture Collection (ATCC) Manassas, VA, USA. HCT-15 and SW-480 cell lines were maintained in RPMI 1640 medium (SAFCO) (Sigma Aldrich, Castle Hill, NSW) supplemented with foetal calf serum (FCS, 10 %) (Hyclone Quantum Scientific, Clayton South, VIC), glutamine (10 mM), 4-2-hydroxyethyl-1-piperazineethanesulfonic acid (HEPES 10 mM) and penicillin (100U/ml)/streptomycin (100 μg/ml) (Sigma Aldrich, Castle Hill, NSW). The Caco-2 cell line was maintained in Dulbecco’s Modified Eagle’s Medium (DMEM) (Sigma Aldrich, Castle Hill, NSW) supplemented with 20%FCS and penicillin (100U/ml)/streptomycin (100 μg/ml), 2 mM/L glutamine, 0.1 mM non-essential amino acids. Cells were grown at 37 °C in 5 % CO_2_ humidified atmosphere.

### Extraction of free fatty acids from oils and fatty acid solution preparation

Krill oil and fish oil (Swisse Wellness Pty Ltd., Victoria, Australia) were purchased from the local Chemist. Free fatty acids were extracted from krill oil and fish oil following the hydrolysis (saponification) method of Salimon et al. [28]. The extracts were dissolved in the Dimethyl Sulfoxide (DMSO) and stored at −20 °C. The final treatment solutions contained 1 % DMSO as solvent. Individual PUFA including EPA, DHA and Arachnoid Acid (AA) were purchased from Nu-Chek-Prep, Elysian, USA. Fatty acid solutions were made up by dissolving the individual fatty acid in ethanol. The final treatment solutions contained < 0.1 % ethanol as solvent.

### Cell proliferation assays

A water-soluble tetrazolium-1 (WST-1) assay kit (Roche Diagnostics GmbH, Germany) was used to determine the proliferative potential of cancer cells. Cells were seeded and cultured at 1 × 10^4^ cells per well in 96-well plates for 24 h, and then treated with individual PUFA solutions for 24, 48 or 72 h; or free fatty acid extract solutions of oils (krill oil or fish oil) for 48 h. All treatments were performed in quadruplicates. For PUFA treatments, three concentrations of each fatty acid were used including 50 μM, 100 μM and 200 μM. 0.1 % ethanol was used as a vehicle control. Additional assays were performed to observe the effects of various EPA concentrations (100 μM, 120 μM, 140 μM, 160 μM, 180 μM and 200 μM) on cell proliferation of HCT-15 cells after 48 h of treatment. The treatment concentrations of free fatty acid extract of oils (krill oil or fish oil) are 0.03, 0.06, 0.12 or 0.24 μL/100 μL well. 1 % DMSO was used as a vehicle control. Non -treated cells were used as a negative control in all experiments. 10 μL WST-1 reagent was added to each well after respective treatment time points and incubated at 37 °C for one hour. Cell proliferation was measured using a microplate reader (Varioskan Flash, Thermo Scientific) at the absorbance of 450 nm.

### Apoptosis assay

Cells (1 × 10^3^ cells/well) were grown in 96-well plates with a clear flat bottom (Corning™ Costar™ 3603, USA) at 37 °C for 24 h. They were then treated by EPA at 100 μM or 200 μM, free fatty acid extract from krill oil at 0.12 μL/100 μM well for 48 h. After removing the media, cells were washed twice by using PBS. Apoptosis/Necrosis was detected using the commercial kit (ab176749, Abcam, Cambridge, England). Cells were examined under the fluorescence microscope (Olympus 1 × 81). The apoptosis was determined by comparing the number of treated cells against the control. Each experiment was performed in quadruplicates, and it was carried out three times for each cell line.

### Mitochondrial membrane potential JC-10 assay

Cells were seeded at 20 × 10^4^ cells/well in the clear bottom 96-well plates (Corning™ Costar™ 3603, USA) to incubate for 24 h at 37 °C. Then the media was added with EPA at 100 μM or 200 μM, or free fatty acid extract from krill oil at 0.12 μL/100 μL well and incubated for 48 h. The mitochondrial membrane potential was measured by using the JC-10 mitochondrial membrane potential assay kit (ab 112134) as per the manufacturer’s instructions (Abcam). In brief, 50 μL of JC-10 reagents was added to each well after the treatment, and incubated for one hour at 37 °C in dark. 50 μL of assay buffer was added to each well. Fluorescence intensity was measured using a microplate reader (Varioskan Flash, Thermo Scientific) at Ex/Em = 485/520 nm and Ex/Em = 540/570 nm. The mitochondrial membrane potential changes were measured as the ratio between aggregate (Em 520 nm) and monomeric forms (Em 570 nm) of JC-10. The increases of the ratio indicate the mitochondrial membrane depolarisation. Quadruplicates were performed for each treatment and two individual experiments were conducted for each cell line.

### Analysis of fatty acid composition in EPA treated cancer cells

HCT-15 cells were seeded at a density of 5 × 10^5^ cells/well in 6-well plates and incubated for 24 h at 37 °C. EPA (100 μM or 200 μM) was added to each well. Each treatment was performed in triplicate. After 48 h incubation, cells were collected into 15 ml Falcon tubes and centrifuged at 1300 rpm for 6 min (Thermo Scientific Haraeus, Megafuge 40). The cell pellets were then used to extract fatty acid methyl esters (FAME) using a modified transesterification method of Lepage and Roy [29]. Briefly, cell pellets were mixed with 2 mL of internal standard, heneicosanoic acid, C21:0 (180 μg) (Nu-Chek Prep, Inc., Elysian, MN) in methanol:toluene (4:1 (v/v)). 200 μL of acetyl chloride was then added and the mixture was incubated for 1 h at 100 °C to form FAME. 5 mL of 6 % potassium carbonate (K_2_CO_3_) in distilled water was then added and the blend was thoroughly vortexed prior to centrifugation at 3000 × *g* (Sigma 3–30 K) for 10 min to separate the layers. The top toluene-rich layer was removed and evaporated to dryness under nitrogen. 200 μL of petroleum ether was added and the resulting FAME was separated by the Gas Chromatography (GC). The GC (Varian Star 3400Cx, Agilent Technologies, CA, USA) was equipped with a SGE BPX 70 capillary column (60 m × 0.25 mm internal diameter, 0.25 μm film thickness) (SGE Analytical Science, Melbourne, Australia), and a flame ionization detector (FID). For each sample, 2 μL was injected in splitting mode (1:10) with helium as the carrier gas. Fatty acids were identified by comparison with a standard FAME mixture, GLC reference standard 403 (Nu-Chek Prep, Inc., Elysian, MN). The composition of each fatty acid was calculated as a percentage of the total amount of fatty acids in the mixture.

### Statistical analysis

All the data were analysed using SPSS 22 software. Mixed model ANOVA was used to determine the significance between treatments. The significance of repeated measure at different time point was analysed using one way ANOVA. *p* < 0.05 was considered significant. The results were expressed as mean ± SD in Table or mean ± SEM in Figures.

## Results

### Effects of PUFA on the proliferation of cancer cells

**Fig. 1:**
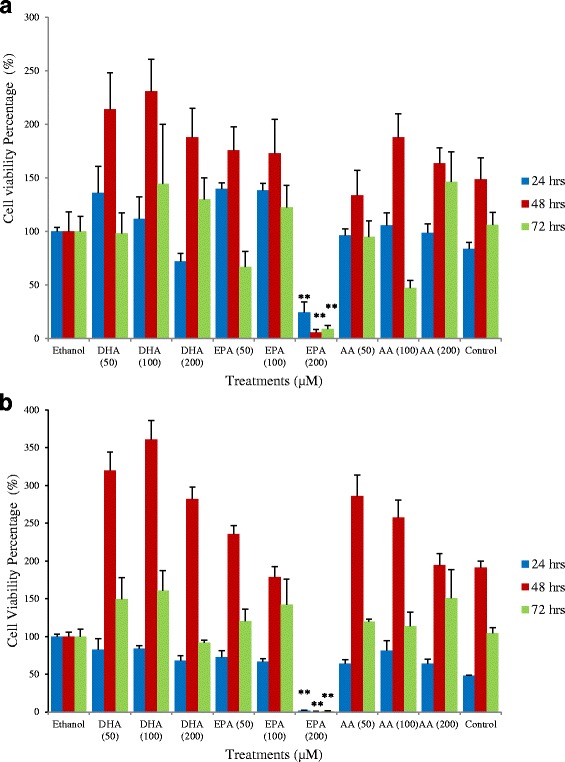
Proliferation of cancer cells after treatment with PUFA. ** indicates a significant difference (*p* < 0.01) compared with ethanol. **a** HCT-15 cell line; **b** SW-480 cell line

Human colon cancer cells of HCT-15 and SW-480 cell lines treated with 200 μM of EPA showed a significant reduction of cell proliferation at all three time points (24, 48 and 72 h) compared to cells treated with ethanol (Fig. 1). It inhibited HCT-15 cell proliferation by 76 ± 0.19 %, 94 ± 0.06 % and 91 ± 0.06 % at 24, 48 and 72 h respectively (Fig. 1a). For SW-480 cells, the inhibitory effect was even more remarkable, with 98 ± 0.01 %, 100 ± 0.02 % and 99 ± 0.01 % reduction in cell proliferation being observed at the three time points respectively (Fig. 1b). However, treatment with lower EPA concentration (50 μM and 100 μM) did not show positive results. Furthermore, no significant effects were recorded after the treatment with DHA or AA. Caco-2 cells did not respond to the treatment of EPA and other two fatty acids at any concentrations and any time points.

**Fig. 2:**
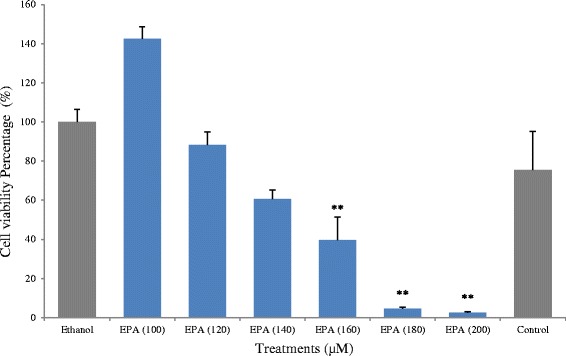
Proliferation of HCT-15 cells after 48 h of treatment with EPA. ** indicates a significant difference (*p* < 0.01) compared with ethanol

To investigate the dose dependent effects of EPA on cell proliferation, four concentrations of EPA solution ranged from 120 μM to 180 μM in addition to 100 and 200 μM were used to treat HCT-15 cells. As shown in Fig. 2, a significant reduction of cell growth (*p* < 0.01) after 48 h of treatment with 160 μM and 180 μM EPA was observed. Cell proliferation has been reduced by 60 % and 95 % respectively compared with 97 % reduction with 200 μM EPA treatment.

### Effects of krill oil extract on the proliferation of cancer cells

**Fig. 3:**
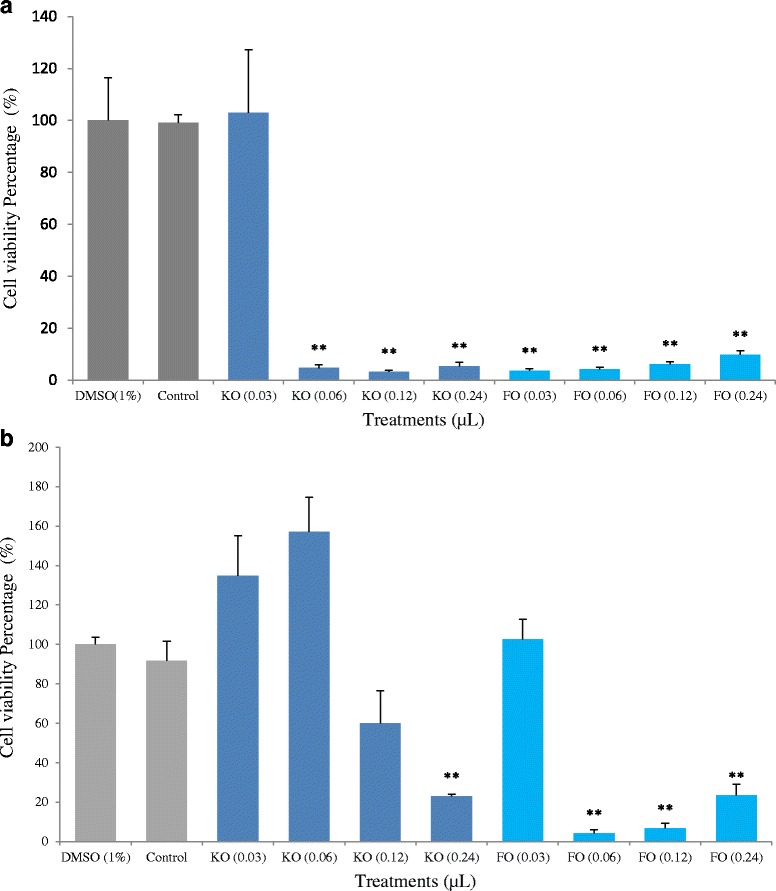
Proliferation of human colon cancer cells after 48 h of treatment with FFA extracts of krill oil and fish oil. ** indicates a significant difference (*p* < 0.01) compared with DMSO control. **a** HCT-15 cells; **b** Caco-2 cells

HCT-15 cancer cells treated with FFA extracted from krill oil for 48 h at the concentrations from 0.06 to 0.24 μL/100 μL significantly reduced cell proliferation compared to DMSO control as shown in Fig. 3a. Similarly, FFA extracted from fish oil also inhibited cell proliferation but started at the relatively lower concentration (0.03 to 0.24 μL/100 μL). The percentage of cell inhibition by krill oil extract ranged from 94 to 96 %, and by fish oil extract from 90 to 96 %. SW-480 cells responded to treatment with FFA extract of krill oil and fish oil in the same manner as HCT-15 cells (results not shown). For Caco-2 cells only the high concentration of FFA extract of krill oil (0.24 μL/100 μL) inhibited cell proliferation significantly (*p* < 0.01), and this may be due to the presence of multidrug resistance-associated proteins in the cells (MRPs) [30]. While lower concentrations of FFA extract of fish oil (0.06 μL/100 μL well) exhibited better inhibitory effects.

### Effects of krill oil FFA and EPA on apoptosis

**Fig. 4:**
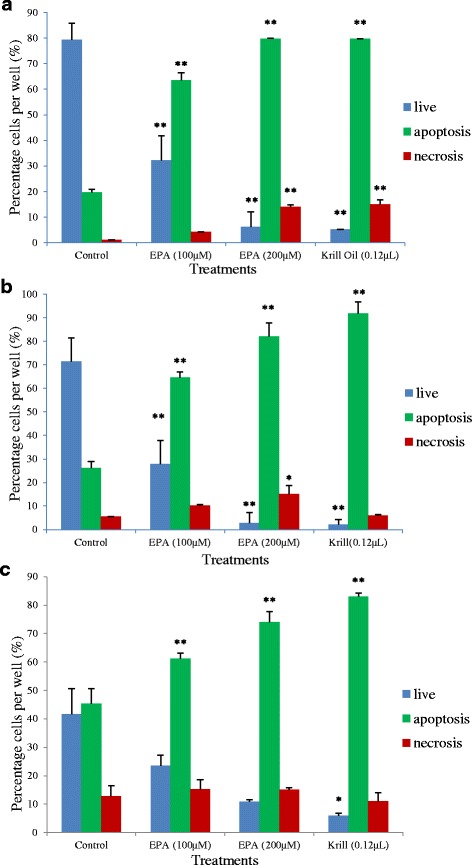
Apoptosis of human colon cancer cells after 48 h of treatment with EPA and FFA extract of krill oil. ** indicates a significant difference (*p* < 0.01) compared with the control. **a** HCT-15 cells; **b** SW480 cells; **c** Caco-2 cells

As shown in Fig. 4, treatment with EPA at 100 and 200 μM and FFA extract of krill oil at 0.12 μL/100 μL well for 48 h resulted in a significantly high apoptosis compared to the control in all three colorectal cancer cell lines. In addition, cell necrosis increased significantly after 48 h treatment with 200 μM EPA and 0.12 μL/100 μL well with FFA extract of krill oil in HCT-15 cells. For SW-480 cells, necrosis was promoted slightly by 200 μM EPA only (*p* < 0.01) while none of the treatments affected the cell necrosis in Caco-2 cells.

### Effects of krill oil FFA and EPA on MMP of cancer cells

**Fig. 5:**
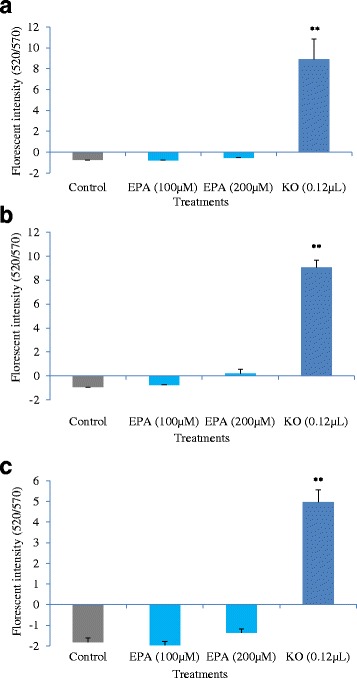
Change in mitochondrial membrane potential (MMP) after treatment with EPA and FFA extract of krill oil for 48 h. ** indicates a significant difference (*p* < 0.01) compared with the control. **a** HCT-15 cells; **b** SW480 cells; **c** Caco-2 cells

The three cell lines HCT-15, SW-480 and Caco-2 were treated with EPA at 100 μM and 200 μM, and FFA extract of krill oil at 0.12 μL/100 μL well for 48 h to measure the mitochondrial membrane potential (MMP) change of the cancer cells. Mitochondrial membrane depolarisation in all three cell lines was significantly higher after treatment with FFA extract of krill oil compared to the control. However, no significant change was observed after EPA treatment (Fig. 5).

### PUFA profile of HCT-15 cells after EPA treatment

HCT-15 cells treated with EPA at concentrations of 100 μM and200μM for 48 h resulted in significant increases in EPA incorporation in the cancer cells (Table 1). EPA treated cells also showed a significantly lower level of AA, especially in cells treated with 200 μM EPA. In addition, 100 μM EPA treatment resulted in a significant increase in DPA (22:5 n-3) level in the cells. Consistently a higher n-3/n-6 ratio was observed in EPA treated cells at both concentrations.

**Table 1:** Fatty acid composition of HCT cells treated by different EPA concentrations

	Control	Ethanol	EPA (100 μM)	EPA (200 μM)
LA (n-6)	0.62 ± 0.04^a^	0.59 ± 0.01^a^	0.47 ± 0.02^b^	0.44 ± 0.01^b^
ALA (n-3)	0.10 ± 0.02	0.09 ± 0.01	0.08 ± 0.01	0.09 ± 0.00
AA (20:4 n-6)	1.46 ± 0.12^a^	1.44 ± 0.06^a^	0.89 ± 0.02^b^	0.74 ± 0.06^b^
EPA (20:5 n-3)	0.25 ± 0.03^a^	0.27 ± 0.05^a^	5.97 ± 0.11^b^	9.57 ± 0.64^c^
DPA (22:5 n-3)	0.71 ± 0.03^a^	0.72 ± 0.01^a^	2.75 ± 0.05^b^	1.54 ± 0.12^b^
DHA (22:6 n-3)	0.76 ± 0.03^a^	0.71 ± 0.02^a^	0.37 ± 0.01^b^	0.36 ± 0.01^b^
EPA + DHA	1.00 ± 0.05^a^	0.98 ± 0.03^a^	6.33 ± 0.11^b^	9.92 ± 0.63^c^
Total n-3	1.81 ± 0.09^a^	1.79 ± 0.04^a^	9.16 ± 0.16^b^	11.55 ± 0.76^b^
Total n-6	2.07 ± 0.14^a^	2.03 ± 0.06^a^	1.36 ± 0.03^b^	1.17 ± 0.06^b^
n3/n6	0.87 ± 0.25^a^	0.88 ± 0.01^a^	6.72 ± 0.11^b^	9.85 ± 0.69^c^

Values expressed as mean ± SD. Values in the same row with different superscript letters indicate the significance (*p* < 0.05)

## Discussion

The main objective of the current study was to investigate the effect of krill oil extract on three human CRC cell lines, HCT-15, SW-480 and Caco-2 cells. The study has demonstrated that FFA extract of krill oil can inhibit cell proliferation significantly in HCT-15 and SW-480 cells, and induced cell apoptosis in all three cell lines. The anti-proliferative effect of krill oil is consistent with the previous studies on colorectal cancer and osteosarcoma cells [8, 26]. Su and co-authors [8] found that the anti-proliferative role of krill oil on osteosarcoma is comparable with 500nM of doxorubicine, a commonly used clinical drug for cancer treatment [31]. The positive outcomes from this study suggest that krill oil may potentially be a useful cancer therapy tool, especially since no adverse effects have been reported with krill oil consumption [23]. Further animal studies are required to confirm the anti-cancer effects of krill oil on CRC in vivo.

It appears likely that the anti-proliferative and pro-apoptotic effects of krill oil extract may be attributed to the EPA in this marine oil. A number of studies have reported the inhibitory effects of EPA, DHA and fish oil on the growth and development of various cancers in vitro and in vivo [6, 13, 32–35]. In vitro studies showed that treatment with EPA or DHA resulted in growth arrest in various human cancer cells. Various mechanisms underlying the anti-cancer effects of EPA have been proposed. These include the inhibition of production of prostaglandin E_2_ (PGE_2_) [36], an eicosanoid derived from the AA, which is associated with cell proliferation, differentiation, apoptosis, angiogenesis and metastasis [37, 38]. EPA competes with AA for the metabolic pathway as they are both catalysed by the same enzymes, and therefore when a high level of EPA is in the cells, more eicosanoids derived from EPA (PGE_3_) will be produced. These eicosanoids inhibit the activity of Akt/pkB kinase expression that reduces the PGE2 level [5] and have contrasting properties to PGE_2,_ such as anti-inflammatory, anti-proliferation and pro-apoptosis [19, 39].

In addition, EPA treatment has been found to induce cell apoptosis via arresting cell cycle in pancreatic cancer cells [40]. This reduced the level of anti-apoptotic bcl-2 protein expression in human leukaemia cells [41]. Chi et al. have shown that EPA induced cell apoptosis activation via p53 dependent Fas/FasL pathway in hepatoma cells [42]. The role of EPA in the modulation of gene expression and activation of caspases involved in cancer cell apoptosis has also been reported [43, 44]. Furthermore, Fukui et al. have shown both in vitro and in vivo that the anti-cancer effects of EPA is associated with the cell death resulted from the accumulation of ROS in the cancer cells [17].

Studies have also shown that EPA and DHA induced apoptosis of colon cancer cells through mitochondrial pathway involving a change of mitochondrial membrane potential (MMP), and the release of cytochrome *c* and other pro-apoptotic factors leading to cell death. In addition, it has been reported that EPA treatment in vitro [17, 45, 46] and FO supplementation in vivo [47, 48] could increase ROS and Ca^2+^ levels in mitochondria and disrupt mitochondrial membrane potential leading to cell apoptosis in various human and rat cancer cells. To investigate whether the pro-apoptotic effect of EPA and krill oil extract in the present study was through mitochondrial pathway, we evaluated the changes in MMP after 48 h of treatment. It was found that there was a significant increase in the depolarization of mitochondria membrane in the cells treated with krill oil extract, and this was correlated with the number of apoptotic cells in the three cell lines (Figs. 4 & 5). However treatment with EPA or DHA did not show significant effect on MMP as krill oil extract (Fig. 5, data on DHA not shown). The results from the current study on MMP suggest that other components of the krill oil extract may play a role in the alteration of MMP, and the EPA induced cell apoptosis was possibly through other mechanisms rather than mitochondrial pathway. Further studies are required to fully understand the mechanisms underlying the pro-apoptotic effects of krill oil extract and whether other mechanistic pathways such as changes in cell cycle, signalling receptors, intracellular Ca^2+^ balance, and induced stress in endoplasmic reticulum etc. also play a role [49].

Studies have shown the anti-cancer effect of DHA on several cancer cell types including colorectal cells [13, 50, 51]. DHA was found to reduce cell proliferation more efficiently than EPA by Schonberg et al. [52]. In the current study, we have not found significant cell inhibition by DHA at concentrations between 50 μM to 200 μM in any of the three cancer cell lines. Our results are at discrepancy with the previous report on SW-480 and Caco-2 cells that showed significant inhibition on cell growth after DHA treatment [53]. Corsetto et al. have reported that DHA inhibits cell growth at the concentrations higher than 200 μM [37]. Schonberg et al. Schonberg SA et al. have found that SW-480 cells are more resistant to DHA treatment [52]. Further study is required to investigate whether higher concentration of DHA can exert anti-cancer effects on HCT-15, SW-408 and Caco-2 cells.

Lipid synthesis is one of the critical functions in cell growth and proliferation. Previous studies have shown that n-3 PUFA induced cell apoptosis is mainly via asymmetric lipid disruption in the cell membrane leading to changes in cell membrane structure, signal pathways and control intracellular homeostasis [54–56]. The results of this study have shown that higher concentration of EPA (200 μM) effectively reduced the cell proliferation in HCT-15 and SW-480 cells. It also highlighted a significant increase in cellular EPA and n-3 fatty acid levels in the cells treated by EPA, and a significant reduction of AA level after treatment with EPA. Consistently, other studies have also shown that EPA supplementation could significantly increase EPA level in the cells, and that suppressed cell proliferation and induced apoptosis [36, 57]. In addition, EPA treatment has significantly increased docosapentaenoic acid (DPA n-3) at the present study. This is in agreement with the results reported by Calviello [57]. This indicates that EPA, after incorporation into cells, maybe further metabolised through elongation to DPA [8]. DPA is also a long chain n-3 PUFA but its health benefits on cancer and other diseases are limited in comparison with EPA and DHA [58].

The results of this study have shown a significant increase in n-3/n-6 ratio after treatment with EPA at both 100 and 200 μM. This is consistent with other studies [59], and it may have clinical relevance. The significantly higher n-3/n-6 ratio has been reported to be associated with a ranged of health benefits including attenuation of carcinogenesis [58]. Mansara et al. also found that a low n-6/n-3 ratio was associated with a decrease in cancer cell proliferation [59].

Our study has demonstrated that the FFA extract of krill oil showed similar anti-proliferative effects as that from fish oil except a slightly more krill oil extract is required to achieve the similar outcomes (Fig. 3). This may be attributed to the higher concentration of EPA in the fish oil. Animal studies are required to compare the anti-cancer effects of krill oil with fish oil given that studies have suggested that krill oil has a higher bioavailability than fish oil due to the unique chemical composition and thus the higher efficiency of fatty acid absorption into the blood [60].

This is the first study to report the pro-apoptotic effects of krill oil on human CRC cells. Although the mechanism is not clear, mitochondrial pathway seems to play a role. It is not known how EPA induced apoptosis at the present study. Krill oil contains a range of FFA including saturated fatty acids (SFA), monounsaturated fatty acids (MUFA) and polyunsaturated fatty acids (PUFA). It could be the synergistic effects of EPA with other fatty acids that resulted in the change of MMP. Taken together, these findings suggest that the pro-apoptotic properties of the krill oil extract may be associated with a complex mechanism, which requires further investigation on the interactions between the individual fatty acids in the oil and evaluation of molecular biomarkers involved in both the intrinsic and extrinsic pathways.

## Conclusion

Krill oil extract has a pro-apoptotic effect on the human CRC cells HCT-15, SW-480 and Caco-2. Its anti-proliferative role was also observed in HCT-15 and SW-480 cells. EPA treatment showed similar health benefits as the FFA extract of krill oil. The FFA extract of krill oil induced the apoptosis of cancer cells may involve a mitochondria-mediated pathway. However, the apoptosis resulted from EPA treatment appears to be independent of the mitochondrial pathway. Further study is required to elucidate the complex mechanisms underlying the anti-cancer properties of krill oil extract. The outcomes of this study showed that krill oil may be protective against CRC as daily nutrient or as a conjunctive therapy for the CRC treatment.

## Abbreviations

AA: Arachnoid acid
ATCC: American tissue culture collection
CRC: Colorectal cancer
DHA: Docosahexaenoic acid
DMEM: Dulbecco’s modified eagle’s medium
DMSO: Dimethyl sulfoxide
DPA: Docosapentaenoic acid
EPA: Eicosapentaenoic acid
FAME: Fatty acids methyl esters
FCS: Foetal calf serum
FFA: Free Fatty Acids
FID: Flame ionization detector
GC: Gas chromatography
HEPES: 4-2- hydroxyethyl −1-piperazineethanesulfonic
LC n-3 PUFA: Long chain n-3 PUFA
MMP: Mitochondrial membrane potential
MRPs: Multidrug resistances-associated proteins
MUFA: Monounsaturated fatty acids
n-6: Omega 6
PBS: Phosphate buffered saline
PGE2: Prostaglandin E2
PGE3: Prostaglandin E3
PUFA: Polyunsaturated fatty acids
ROS: Reactive oxygen species
SD: Standard deviation
SEM: Standard error of mean
SFA: Saturated fatty acids
WST-1: Water soluble tetrazolium-1
